# *miR-140* and *miR-196a* as Potential Biomarkers in Breast Cancer Patients

**DOI:** 10.31557/APJCP.2020.21.7.1913

**Published:** 2020-07

**Authors:** Arman Shahabi, Behrooz Naghili, Khalil Ansarin, Vahid Montazeri, Nosratollah Zarghami

**Affiliations:** 1 *Infectious and Tropical Diseases Research Center, Tabriz University of Medical Sciences, Tabriz, Iran. *; 2 *Cell Therapy and Regenerative Medicine Comprehensive Center, Kerman University of Medical Sciences, Kerman, Iran. *; 3 *Department of Clinical Biochemistry and Laboratory Medicine, Faculty of Medicine, Tabriz University of Medical Sciences, Tabriz, Iran. *; 4 *Department of Thoracic Surgery, Faculty of Medicine, Tabriz University of Medical Sciences, Tabriz, Iran. *

**Keywords:** Biomarkers, miR-140, miR-196a, quantitative PCR, breast cancer

## Abstract

**Objective:**

*MiR-140* and* miR-196a* were known to be correlated with cancer diagnosis and prognosis. The current study aimed at the analysis of *miR-140* and *miR-196a *expression patterns and their clinical significance for breast cancer (BC) patients.

**Methods:**

Differentially expressed *miR-140 *and *miR-196a* were examined via quantitative PCR in 110 cases of BC and their adjacent non-tumor (ANT) tissues.

**Results:**

The results indicated that *miR-140* and *miR-196a*, respectively, notably decreased and increased expression in BC samples in comparison with ANT (p<0.001). Reduced *miR-14*0 expression was also related to Lymph node metastasis (LNM, P= 0.023) and stage (P = 0.009). Additionally, Receiver Operating Characteristics (ROC) analysis illustrated that *miR-140* had a significant diagnostic accuracy for stage and LNM of BC patients. We also discovered a strong negative correlation between *miR-196a* expression with histological grade (P = 0.038), LNM (P = 0.012) and stage (P = 0.001).

**Conclusion:**

Overall, exploring the *miR-140* and *miR-196a* profiles not only can statistically different among BC and ANT samples, but it is also expected to become potential BC biomarkers.

## Introduction

As the most predominant malignancies in females, breast cancer (BC) accounts for the number one cause of cancer mortality worldwide among women (Jafari-Gharabaghlou et al., 2018; Javidfar et al., 2018). The relationship between genes and environmental risk factors associated with the etiopathology of various tumors (Rudolph et al., 2016). Recently, many studies have been made aiming to identify the connection between genetic alterations of protein-coding genes and mechanisms of carcinogenesis (Chakravarthi et al., 2016). But latest studies have supported the potential role of noncoding RNAs in tumorigenesis, in which miRNAs recognized as new players in cancer pathobiology (Sheervalilou et al., 2016; Mohammadian et al., 2017a). microRNAs (miRNAs/miRs) are short (about 18–23 nucleotides), conserved, noncoding regulatory RNAs that negatively regulate gene expression based on targeting of the mRNA 3′-untranslated regions (3′-UTR) (Mohammadian et al., 2016b; Norouzi et al., 2019). Computational approaches report that miRNA-mediated the activity of about 30% of all human genomes, in which each miRNA on average potentially interact with >100 of mRNA targets (Mohammadian et al., 2016a; Sheervalilou et al., 2020). The biological function of miRNAs as either tumor suppressors or oncogenes related to their target mRNA (Bertoli et al., 2015; Shahabi et al., 2019). Given that the particular miRNA signatures reported in many tumor types, so, logically, the alterations of miRNA profiles would be promising diagnostics and prognostic cancer biomarkers (Mohammadian et al., 2017b; Rupaimoole and Slack, 2017). The dysregulation of miRNA profiles may contribute to the malignant transformation such as cancer initiation, progression, and therapeutic resistance (O’Bryan et al., 2017). At the moment, numerous investigations have illustrated that *miR-140* and *miR-196a* expression frequently are altered in human cancers tissues, which proposes that they play key roles in tumorigenesis (Schimanski et al., 2009; Green et al., 2015). 

Recent studies have demonstrated that *miR-140* expression mediated phenotypic alterations of tumor cells (Fang et al., 2017). As an example, *miR-140* expression significantly represses the growth and BC stem cell self-renewal potential (Li et al., 2013) and effectively prevents tumorigenicity of glioblastoma, osteosarcoma and non-small cell lung carcinoma (NSCLC) (Liu et al., 2016; Ji et al., 2018; Yang et al., 2018). Indeed, numerous published studies have displayed that the *miR-140* may serve as tumor suppressor miRNA and its replacement has been indicated as a novel therapeutic strategy among different cancer types (Fang et al., 2017; Li et al., 2018). miR-196a belongs to the homeotic genes (HOX) family which has different function in various types of cell lines and tissues (Chen et al., 2011). Several recent profiling studies of miRNAs reported that upregulated miR-196a expression was significantly linked to tumor progression (Sun et al., 2012; Jin et al., 2016). Besides, numerous studies illustrated that miR-196a has significant effects on the growth, differentiation, and metastasis of tumor cells based on the regulation of specific genes (Papaconstantinou et al., 2012; Jiang et al., 2018). These investigations permit us to simultaneously evaluate the miR–196a and miR–140 expression profiles in BC tissues and their matched adjacent non-tumor (ANT) tissues, and to demonstrate their clinicopathological significance. Our results support the interpretation that upregulation of tumor suppressor miR–140 and downregulation of oncogene miR-196a may be an intriguing possibility for future BC treatment.

## Materials and Methods

*Patient and clinical specimens*

All specimens (BC and ANT tissues) were acquired from 110 patients admitted in Noor Nejat Hospital (Tabriz, Iran) and then transferred to −80°C before further analysis. Current research underwent ethical review and was applied through the ethics committee of Tabriz University of Medical Sciences.

*Real-time qRT–PCR for miRNAs*

The relative levels of miR–140 and miR–196a in both BC and ANT samples were analyzed by Real-time quantitative PCR (qPCR) method (Jeddi et al., 2019). The tissues were homogenized using a tissue homogenizer and total RNA was isolated from the homogenized tissue following the Exiqon’s miRCURY™ RNA Isolation Kit (Exiqon, Denmark) and Phenol/Chloroform methods. Total RNA concentrations were investigated by using NanoDrop 2000 (Thermo Scientific, Waltham, MA). Also, the integrity of total RNA was investigated by agarose gel electrophoresis on 1.5% agarose gel containing GelRedTM (Biotium). PCR reactions for quantifying *miR-140* and *miR-196a* were performed in triplicate by LNA Universal RT miRNA PCR kit (Exiqon, Denmark). qPCR protocol was: 95°C pre-denaturation step for 10 min, 95°C 40 cycles of denaturation step for 15 seconds, annealing, and synthesis steps at 60°C for 1 min. The relative expression levels of *miR-140* and *miR-196a* were normalized by U6 and the results were calculated based on the 2^−ΔΔCt^ method (Livak and Schmittgen, 2001). The sequence and details of PCR primer for mature *miR-140*, miR-196a, and U6 are shown in Table 1.

*Data analysis*

Mann-Whitney U test was applied for the evaluation of statistical differences in tissue miRNAs expression between BC and ANT groups. Chi-square test (*χ*^2^) was to measure the relationship among both *miR-140* and miR196a expression levels and clinical parameters. Receiver operating characteristic curves (ROC curves) were considered for investigation mentioned miRNAs as two biomarkers. Statistical analyses were investigated by the SPSS^®^ software (SPSS, Version 16.0; SPSS Inc, Chicago, IL) or GraphPad Prism Software (version 6, GraphPad software, lnc., San Diego, CA, USA).

## Results

*Patient characteristics*

From April 2016 to July 2018, 110 female BC patients were recruited for the present study. The clinicopathological information of all participants is indicated in Table 2. The mean age was 46 years and the range of 27–65 years. 

*Differential miR-140 and miR-196a expression levels in BC and ANT tissues*

**Figure 1:**
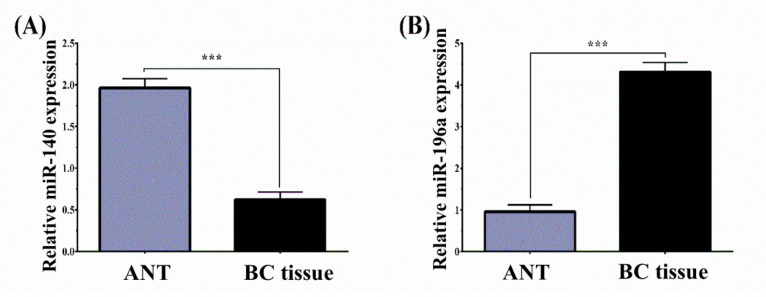
MiR-140 and miR-196a Expression Levels of 110 BC and ANT Tissues Normalized to U6 Analyzed by Using qRT-PCR. The results represent that (A) miR-140 was downregulated in BC compared to that in ANT group (BC vs. ANT: 0.623 ± 1.07 vs 1.74 ±1.393, P< 0.001) and (B) miR-196a was upregulated in BC compared to that in ANT group (BC vs ANT: 4.31 ± 2.55 vs 2.77 ± 2.45, ***P< 0.001). BC, Breast cancer; ANT, Adjacent non‐tumor; qRT-PCR, Real-Time Quantitative Reverse Transcription PCR

Using the RT-qPCR method, the relative level of *miR-140* and *miR-196a* expression was assessed in BC and ANT tissues. Our findings illustrated that the relative level of *miR-140* in BC tissues significantly was significantly downregulated than that of the ANT tissues (BC vs. ANT: 0.623 ± 1.07 vs 1.74 ±1.393, P< 0.001) (Figure 1A). However, the expression of miR-196a was higher in BC patients than that in ANT (BC vs ANT: 4.31 ± 2.55 vs 2.77 ± 2.45, P< 0.001) (Figure 1B). To investigate the correlation of these miRNAs with clinicopathologic features, we classified BC patients into *miR-140*-low/high and miR-196a-low/high expression groups according to the median value of *miR-140* and *miR-196a* expression.

*Correlation between downregulated miR-140 expression and clinical characteristics*

**Figure 2:**
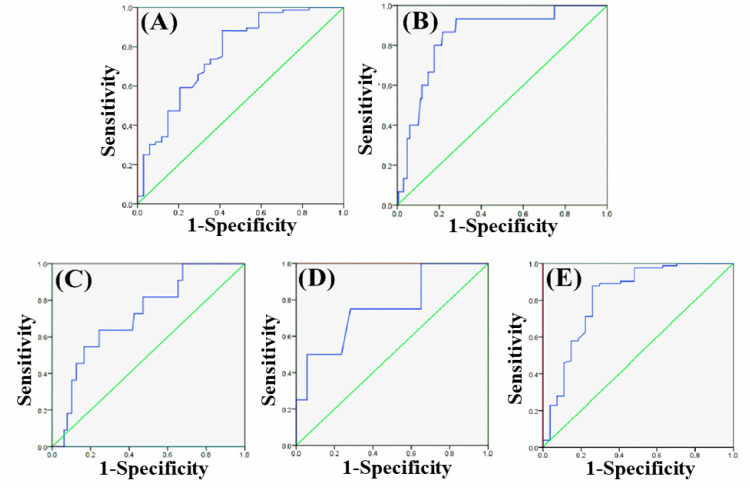
ROC Curve Analysis of the Expression *miR-140 *and *miR-196a* to Detect BC. Expression of miR-140 was an applicable biomarker for prediction of occurrence of LNM (A), and stage (B). Moreover, ROC curve analysis revealed the potential of the miR-196a level to predict histological grade (C), the occurrence of LNM (D) and stage (E), and in BC. BC, Breast cancer; LNM, Lymph node metastasis; ROC, Receiver operating characteristics

Correlation of *miR-140* relative expression level with clinical criteria is presented in Table 2. Low *miR-140* expression was positively associated with Lymph node metastasis (LNM, P= 0.023) and stage (P = 0.009) in BC patients. But, correlations between *miR-140* expression and age, histological grade, estrogen receptor (ER) expression, progesterone receptor (PR), and human epidermal growth factor receptor 2 (HER2) were not statistically significant. Additionally, the ROC curve was plotted to investigate the miRNA diagnostic power cancer detection. According to the ROC curve, *miR-140* had the ability only to predict stage and LNM occurrence in BC patients (Table 3). At an appropriate cut-off value, *miR-140* superior performance in predicting the LNM occurrence (AUC=0.765, P=0.001) and stage (AUC=0.847, P<0.001). The specificity and sensitivity values, respectively, were 77% and %, 68, and 95%, and 87% (Figure 2A, B). 

*Correlation between upregulated miR-196a expression and clinical characteristics*

As showing in Table 2, high miR-196a expression was closely linked with histological grade (P = 0.038), LNM (P = 0.012) and stage (P = 0.001) in BC patients. However, no remarkable associations between the miR-196a expression and age, ER/PR, and Her2 status were evident. Based on the ROC findings, miR-196a was an important predictor of histological grade, LNM, and stage in BC patients (Table 3). At an appropriate cut-off value, miR-196a superior performance in predicting the histological grade (AUC=0.718, P=0.017), LNM (AUC=0.758, P=0.010) and stage (AUC=0.820, P<0.001). The specificity and sensitivity values, respectively, were 72% and 65%, 74% and 70%, and 88% and 81% (Figures 2C, D, E).

## Discussion

Tumor markers are highly important for early detection, outcome prediction, and selection of effective treatment strategies (Mehta et al., 2010). As involved in different cancer biological processes, miRNA studies have become imperative for the detection of miRNA profiles-based prognosis, diagnosis, and theranostics biomarkers (Sheervalilou et al., 2020; Wang et al., 2018). Altered expression of miRNAs provides crucial information about molecular signatures of disease status without the performance of biopsy (Lu et al., 2005; Ayub et al., 2015). The objective of our research was to clarify the prognostic significance of *miR-140* and *miR-196a* in 110 Iranian women with BC and to describe their possible relationship with clinicopathological criteria. Our data exhibited that the *miR-140* and *miR-196a* levels in BC samples were notably lower and higher than those in corresponding ANT tissues. The reduced level of miR–140 was significantly relevant to LNM and stage. Besides, ROC results illustrated that the expression of miR–140 was an applicable molecular biomarker for the prediction of LNM occurrence and stage in BC patients. Based on prior studies results, *miR-140* considered being one of the importantly reduced miRNAs in human cancers such as BC. For instance, Zhou et al., (2019) found that high levels of *miR-140* expression could significantly suppress BC cell growth and migration. In another study, Lu et al., (2017) also found similar results that the level of miR–140 was negatively related to the patient’s clinical-grade and tumor metastasis. Furthermore, both gain and loss of function studies by Yuan et al., (2013) revealed that *miR-140* represses the NSCLC metastasis. Also, the high expression of *miR-145* significantly reduced lung cancer growth in nude mice models. Other studies also reported that *miR-140* was downregulated in cell lines and human tumor tissues (Liu et al., 2016; Yan et al., 2017). According to our study, an inverse association also found between the miR-196ea expression profile and histological grade, LNM, and stage. Besides, ROC curve analysis indicated the potential of miR–196a level to predict histological grade, LNM occurrence, and stage in the BC. Based on the tumor tissue and the cell type, miR-196a has oncogenic or tumor-suppressor functions. Few reports have revealed the miR-196 family function as a tumor suppressor through negatively regulating the expression of oncogenes. For instance, Cao et al., (2018) indicated that miR-196a level was considerably lower in clinical osteosarcoma tissues than normal controls, and the osteosarcoma cell progression and migration abilities were inhibited by miR-196 overexpression. Likewise, in melanoma, miR-196a inhibits the processes of proliferation and metastasis through the negative regulation of transcription factor HOXC8 (Mueller and Bosserhoff, 2011). However, several other studies (e.g. Liu et al. (Liu et al., 2012); Hou et al., (2014); Yang et al., (2016); Milevskiy et al., (2019) have examined and reported the significant roles of miR-196a in promoting proliferation, migration and invasion of NSCLC, cervical, epithelial ovarian and ER+ BC cell lines and malignant tissues in comparison with healthy tissues. 

Our findings recommend that both *miR-140* and miR-196a are promising biomarkers for the diagnosis and management of BC patients. Nevertheless, the shortcomings of the present study were that we could not investigate the correlation between miRNA expression and the survival status because it needs a 5-year follow-up of BC patients.

In conclusion, briefly, the results exhibited that both *miR-140* and *miR-196a* may be potential biomarkers for determining the BC diagnosis and prognosis, which may serve, respectively, as a tumor-suppressing and -promoting gene. However, more research is required to determine the *miR-140* and *miR-196a *molecular mechanisms in the BC progression.

**Table 1:** Primer Sequences

Objective genes	Primer sequence
*miR-140*	Forward: 5′-GAGTGTCAGTGGTT TTACCCT-3′
	Reverse: 5′-GCAGGGTCCGAGGTATTC-3′
*miR-196a*	Forward: 5′-CGTCAGAAGGAATGATGCACAG-3′
	Reverse: 5′-ACCTGCGTAGGTAGTTTCATGT-3′
*U6*	Forward: 5'-CTCGCTTCGGCAGCACATATACT-3'
	Reverse: 5'-ACGCTTCACGAATTTGCGTGTC-3'

**Table 2:** Relationship between *miR-140* and *miR-196a* Expression with Clinicopathological Parameters of BC Patients. (n = 110). BC, Breast cancer

Clinical pathological criteria			miR–140 Expression			miR–196a Expression		
			Low 53 (%)	High 57 (%)	*P*-value	Low 49 (%)	High 61 (%)	*P*-value
Age	<50	75	32	43	NS	29	46	NS
	>50	35	21	14		20	15	
histological grade	I	21	9	12	NS	8	13	0.038
	II	79	38	41		35	44	
	III	10	6	4		6	4	
Progesterone receptor	negative	34	20	14	NS	18	16	NS
	positive	76	33	43		31	45	
Estrogen receptors	negative	27	12	15	NS	9	18	NS
	positive	83	41	42		40	43	
HER2	negative	99	50	49	NS	45	54	NS
	positive	11	3	8		4	7	
lymph node metastasis	negative	46	21	25	0.023	18	28	0.012
	positive	64	32	32		31	33	
Stage	I	16	7	9	0.009	7	9	0.001
	II	72	37	35		35	37	
	III	18	8	10		7	11	
	IV	4	1	3		0	4	
Tumor size	<20mm	39	16	23	NS	14	25	NS
	20-49mm	61	32	29		31	30	
	>50mm	10	5	5		4	6	

**Table 3:** AUC of ROC Curve Corresponding to the Diagnostic *miR-140* and *miR-196a* Values in BC. AUC, Area under the curve; ROC, Receiver operating characteristics; BC, Breast cancer

	*miR–140* Expression				*miR–196a* Expression			
Parameter	AUC	Standard Error	95 % C.I	*P*-value	AUC	Standard Error	95 % C.I	*P*-value
Histological grade	0.635	0.09	0.458–0.812	0.143	0.718	0.072	0.576–0.859	0.017
lymph node metastasis	0.765	0.051	0.665–0.866	0.001	0.758	0.061	0.671–0.845	0.01
Stage	0.847	0.049	0.751–0.942	<0.001	0.82	0.054	0.714–0.927	<0.001
